# Some Account of the Medicinal Properties of a Bark Lately Procured from South America

**Published:** 1789

**Authors:** J. Ewer

**Affiliations:** Physician in Trinidad


IV. *Some Account of the medicinal Properties of a Bark lately procured from South America. By* J. Ewer, *M. D. Phyfician in Trinidad. Communicated in a Letter to Dr.* Simmons *by Meffrs.* Taylor *and* Davy, *Druggifts in London.*

*To Dr.* Simmons.

Sir,

FROM the very favourable account we have received of the medicinal properties of a bark which has lately been fent to us, we are induced to trouble you with a letter in its favour from a Phyfician of eminence in the Weft Indies, hoping by this means the Public may become acquainted with a valuable remedy, and have an opportunity of judging how far it merits the encomiums which he gives it.

This bark is known by the name of *Cortex Anguftura*. We are able to fupply with it any

 perfons perſons who may wiſh to give it a trial; and if its good effects ſhould be confirmed by experience here, we ſhall take care to procure an ample and regular ſupply of it.

We remain, Sir,

Your obliged and obedient Servants,

Taylor and Davy.

*Little Britain,*

March 24, 1789.

---

*To Meſſieurs* Taylor *and* Davy.

Gentlemen,

I have ordered to be ſhipped to you from Grenada a quantity of bark which has been brought hither by the Spaniards from Anguſtura in South America, and has acquired great reputation here in all thoſe caſes in which we have been accuſtomed to employ the Peruvian bark, over which it has this advantage, that a ſmaller doſe of it will produce the ſame effect.

With reſpect to its ſenſible properties, it is exceedingly bitter, and leaves a pungent heat in the mouth; it has a light aromatic ſmell; its exterior ſurface is almoſt white, and its interior of a light brown colour. In many caſes of

 fever fever I have used it with success; and in two or three cases I have found a single dose have a striking good effect. As an external application in a putrid fever, I had lately a strong proof of its efficacy. In this case the patient's skin was of a greenish yellow colour, and had a number of large livid spots; he had hiccough and a vomiting of dark-coloured dissolved blood; a mortification had begun in his throat, and his strength was exceedingly exhausted. As neither the Peruvian bark nor any thing else could be retained on the stomach, and as I did not think it right to trust to this bark given by injection alone, I ordered flannel wetted with a strong warm decoction of this bark to be wrapped round his body and extremities, and kept constantly wet. On seeing him a few hours after I was agreeably surprised on finding the livid spots removed, the greenish colour of the skin gone off, and the hiccough and vomiting ceased. He could now keep the Peruvian bark, mixed with a strong infusion of this bark, on his stomach, and soon after was able to take any kind of nourishment. As he complained of being uneasy at laying so long in wet clothes, his attendants discontinued the use of the fomentation, and in a few hours the greenish yellow colour of the skin and and the livid ſpots returned, but neither the vomiting nor hiccough. The fever was now increaſed conſiderably, and his ſtrength diminiſhed. The fomentation was again had recourſe to with the ſame or even better ſucceſs than before; for, when it had been applied a few hours, he was ſo much better as to be able to get out of bed and ſit up without aſſiſtance: it was, however, diſcontinued again for the ſame reaſon as at firſt, and the ſymptoms again returning, he died in two days.

In this caſe it evidently appears that beneficial effects attended its uſe; and it is much to be regretted that the obſtinacy of the patient prevented the continuance of a remedy which, even under the circumſtances I have mentioned, afforded ſuch a rational hope of ſucceſs.

This bark is uſed with great advantage among our ſlaves here as a bitter and ſtomachic. It has alſo done great ſervice in the dyſentery, a diſeaſe in this country both frequent and fatal.

I am, Gentlemen,

Your moſt obedient, humble Servant,

J. Ewer.

*Trinidad,*

Auguſt 20, 1788.

